# The Key *Trichoderma*-Induced Gene Encoding a DUF568 Domain-Containing Protein Mediates Defense Responses in Wheat

**DOI:** 10.3390/plants15172705

**Published:** 2026-09-03

**Authors:** Junchang Li, Yingying Jin, Yingxue Wang, Tingyu Wang, Huifang Zhang, Yongjing Ni

**Affiliations:** 1 Henan Province Key Laboratory of Efficient Crop Production and Food Quality Safety, Zhoukou Normal University, Zhoukou 466001, China; meekie@163.com; 2 Modern Agricultural Industry Research Institute, Henan Zhoukou National Agricultural High-Tech Industry Demonstration Zone, Zhoukou 477100, China; 15939236787@163.com (Y.J.); 15936553190@163.com (Y.W.); wangtingyu101213@163.com (T.W.); 3 Wheat Technology Innovation Center, Henan Zhoukou Agricultural High-Tech Industry Demonstration Zone, Zhoukou 477100, China; 4 Shangqiu Academy of Agricultural and Forestry Sciences, Shangqiu 476000, China; nyj317@163.com; 5 The Suihuang Laboratory, Shangqiu 476000, China

**Keywords:** wheat, *Trichoderma*, DUF568, defense response, disease-resistance

## Abstract

Genes encoding DUF568 domain-containing proteins participate in plant stress adaptation. To elucidate the functional role of DUF568 domain-containing genes in *Trichoderma*-induced wheat defense responses against wheat Fusarium crown rot, we performed a genome-wide identification and characterization of the *TaDUF568* gene family in hexaploid wheat (*Triticum aestivum* L.). In this study, a total of 33 *TaDUF568* family genes were systematically identified and characterized at the genome-wide level, exhibiting uneven chromosomal distribution and diverse physicochemical properties. Phylogenetic, structural, and collinearity analyses revealed conserved family characteristics among monocot species. Segmental duplication was verified as the primary driver of gene family expansion. Expression profiling revealed divergent tissue-specific expression patterns among *TaDUF568* family members, among which *TaDUF568.18* was strongly induced by *Trichoderma* M2. Subcellular localization assays confirmed that *TaDUF568.18* is a plasma membrane-localized protein. Functional validation via stable transgenes demonstrated that overexpression of *TaDUF568.18* restricted lesion expansion, improved agronomic traits, and enhanced disease resistance. This study is the first to characterize the wheat DUF568 family and confirm that *TaDUF568.18* (annotated as *TaAIR12*) acts as a positive regulator of *Trichoderma*-mediated wheat defense, providing a valuable gene resource for wheat disease-resistance breeding.

## 1. Introduction

Domains of unknown function (DUFs) constitute a large group of structurally conserved protein domains with unvalidated biological functions, representing a major fraction of unannotated coding sequences in plant genomes and serving as untapped genetic resources for deciphering plant developmental regulation and stress adaptation mechanisms [1,2]. As a typical plant-specific conserved domain, proteins containing DUF domains are widely distributed in terrestrial plants and have attracted increasing research attention in recent years [3]. With the rapid advancement of whole-genome sequencing and functional genomics, an increasing number of DUF-containing gene families have been demonstrated to participate in diverse essential biological processes in plants [4,5,6].

As of 2026, as documented in the Pfam database, over 4000 DUF domain families have been identified across 150 species [7], and accumulating functional evidence indicates that plant DUF genes are extensively involved in biotic defense responses, abiotic stress tolerance, phytohormone signal transduction, secondary metabolism, and cellular homeostasis modulation [1]. Notably, numerous crop-based studies have confirmed that DUF family genes act as pivotal regulatory hubs in response to pathogen invasion and adverse environmental stimuli, making them valuable target resources for molecular genetic improvement and stress-resistance breeding of crops [8,9]. The *DUF568* family (PF04526) is a distinctive plant-specific conserved DUF subfamily that is exclusively present in terrestrial plants [10]. Compared with well-characterized DUF families such as *DUF221*, *DUF569* and *DUF4228*, which have been comprehensively studied in multiple plant species, the evolutionary characteristics, structural conservation, and biological functions of the *DUF568* family remain largely unclear [10,11,12,13]. Current research on *DUF568* genes is still limited to preliminary functional explorations in model and crop plants, such as rice [10]. In *Arabidopsis thaliana*, *AIR12* (*Auxin induced in root cultures*), a typical *DUF568* family member encoding an auxin-responsive protein, is localized to the plasma membrane and apoplast, and participates in auxin-dependent root morphogenesis and the regulation of basal immune defense [14,15]. In rice, homologous DUF568 genes exhibit significantly altered expression under salt and drought treatments, indicating their potential regulatory roles in plant abiotic stress tolerance [10].

In recent years, *Trichoderma* spp. have served as efficient, eco-friendly biocontrol agents against crop fungal pathogens, functioning through mycoparasitism, antibiosis, nutrient competition, and activation of host systemic resistance [16,17]. *Trichoderma* colonization triggers extensive host transcriptional reprogramming, activating defense and antioxidant pathways to enhance plant stress tolerance [18,19]. RNA-seq transcriptomics has become a core tool to unravel the molecular interactions between *Trichoderma* and host plants [20]. Transcriptomic studies have verified that *Trichoderma* treatment remodels host defense, hormone signaling and cell wall metabolism pathways in multiple crops [21,22,23]. Importantly, *Trichoderma*-induced transcriptional changes provide a reliable strategy for excavating uncharacterized stress-resistant genes in wheat, facilitating the functional exploration of unknown gene families [24].

This study clarifies the evolutionary and functional features of the wheat DUF568 family, provides candidate genes for wheat disease resistance breeding, and establishes a theoretical basis for dissecting DUF568-mediated mechanisms underlying plant stress adaptation.

## 2. Results

### 2.1. Identification of DUF568 Gene Family Members in Wheat

A total of 33 DUF568-containing gene family members were systematically identified from the whole genome of wheat (Table 1) and designated as *TaDUF568.1* to *TaDUF568.33* according to their chromosomal order. All identified genes were anchored to specific wheat chromosomes, including 2A, 2B, 2D, 4A, 4B, 4D, 5A, 5B, 5D, 7A, 7B and 7D, exhibiting uneven chromosomal distribution. Notably, chromosome 4 harbored the largest number of TaDUF568 members, with 12 genes located on the 4A, 4B and 4D subgenomes; chromosomes 2 and 7 contained 6 and 5 members, respectively, and chromosome 5 contained 10 members.

The encoded TaDUF568 proteins exhibited divergent physicochemical properties. The amino acid length of TaDUF568 proteins ranged from 122 aa (TaDUF568.19) to 416 aa (TaDUF568.4), with molecular weights ranging from 12.75 kDa to 44.85 kDa. The isoelectric point (pI) values ranged widely from 4.78 to 10.12. Most TaDUF568 proteins exhibited alkaline properties with pI values higher than 7.0, whereas only a few members were acidic or neutral. These characteristics imply functional divergence within the TaDUF568 family and lay a foundation for further functional characterization.

### 2.2. Phylogenetic Analysis of DUF568 Gene Family Members

**Figure 1:**
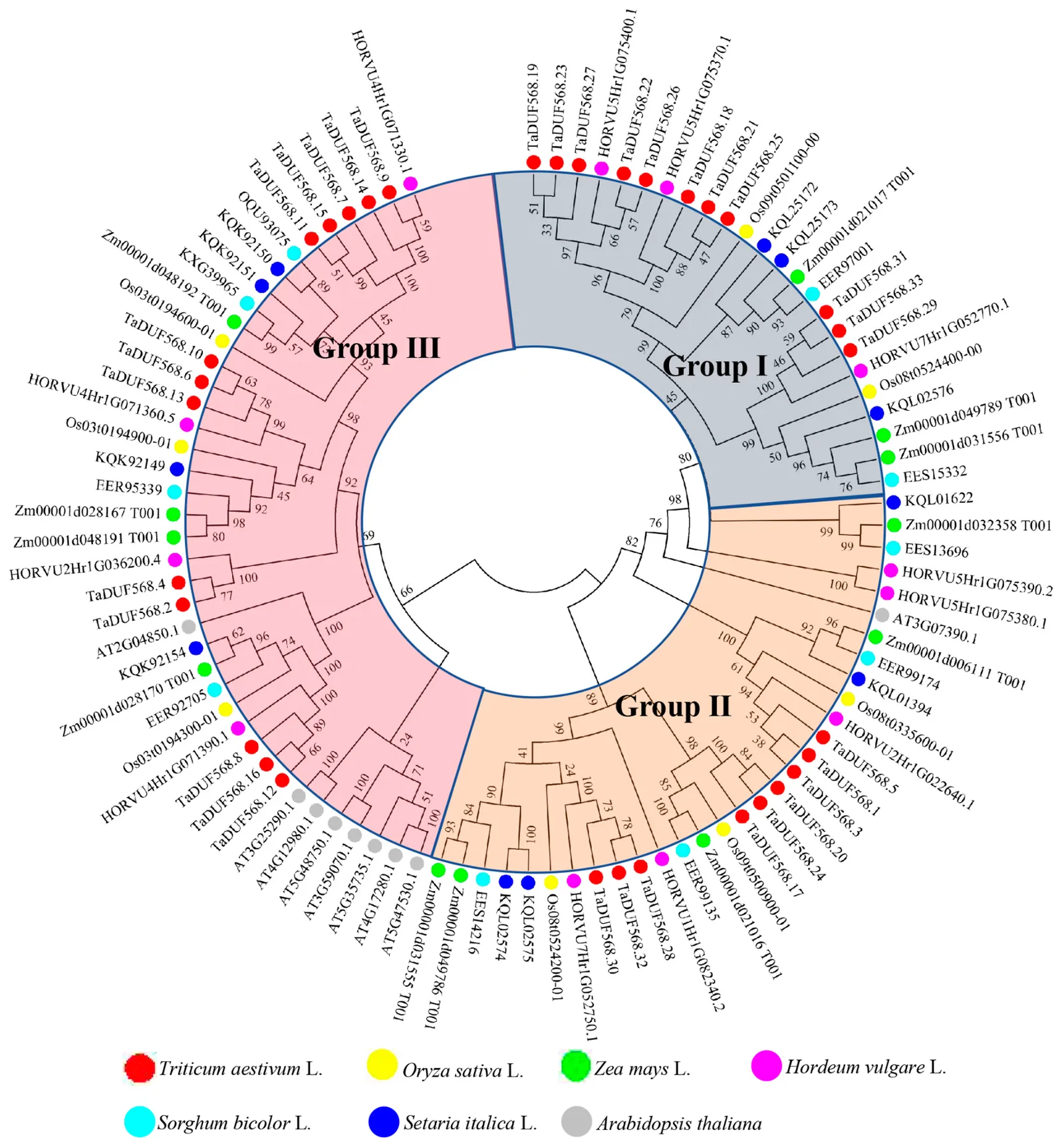
Phylogenetic analysis of wheat *DUF568* gene family members. The different colored solid circles indicate DUF568 proteins. Among them, red circles represent DUF568 proteins in *T. aestivum*; yellow circles represent DUF568 proteins in *O. sativa*; green circles represent DUF568 proteins in *Z. mays*; purple circles represent DUF568 proteins in *H. vulgare*; light blue circles represent DUF568 proteins in *S. bicolor*; blue circles represent DUF568 proteins in *S. italica*; gray circles represent DUF568 proteins in *A. thaliana*. The different colored arcs denote different groups (or subgroups) of DUF568 proteins.

A phylogenetic analysis of the DUF568 gene family was performed using full-length protein sequences from *Triticum aestivum* (Ta), *Oryza sativa* (Os), *Zea mays* (*Zm*), *Hordeum vulgare* (Hv), *Sorghum bicolor* (Sb), *Setaria italica* (Si), and *Arabidopsis thaliana* (At) to clarify their evolutionary relationships. The unrooted neighbor-joining tree classified all identified DUF568 proteins into three distinct groups (Group I, II, and III), with robust bootstrap support for most clades (Figure 1, Table S1). Group I contained 11 wheat DUF568 members, while Group II and III harbored 9 and 13 members, respectively. Notably, wheat DUF568 genes were distributed across all three groups, and most exhibited closer phylogenetic relationships with their orthologs in *H. vulgare* and other monocot species, consistent with their taxonomic classification. Several wheat DUF568 proteins formed species-specific subclades within each group, suggesting lineage-specific expansion events during wheat evolution. *A. thaliana* DUF568 members were restricted to Groups II and III and absent from Group I, indicating distinct evolutionary patterns between monocot and dicot DUF568 homologs.

### 2.3. Sequence Analysis

**Figure 2:**
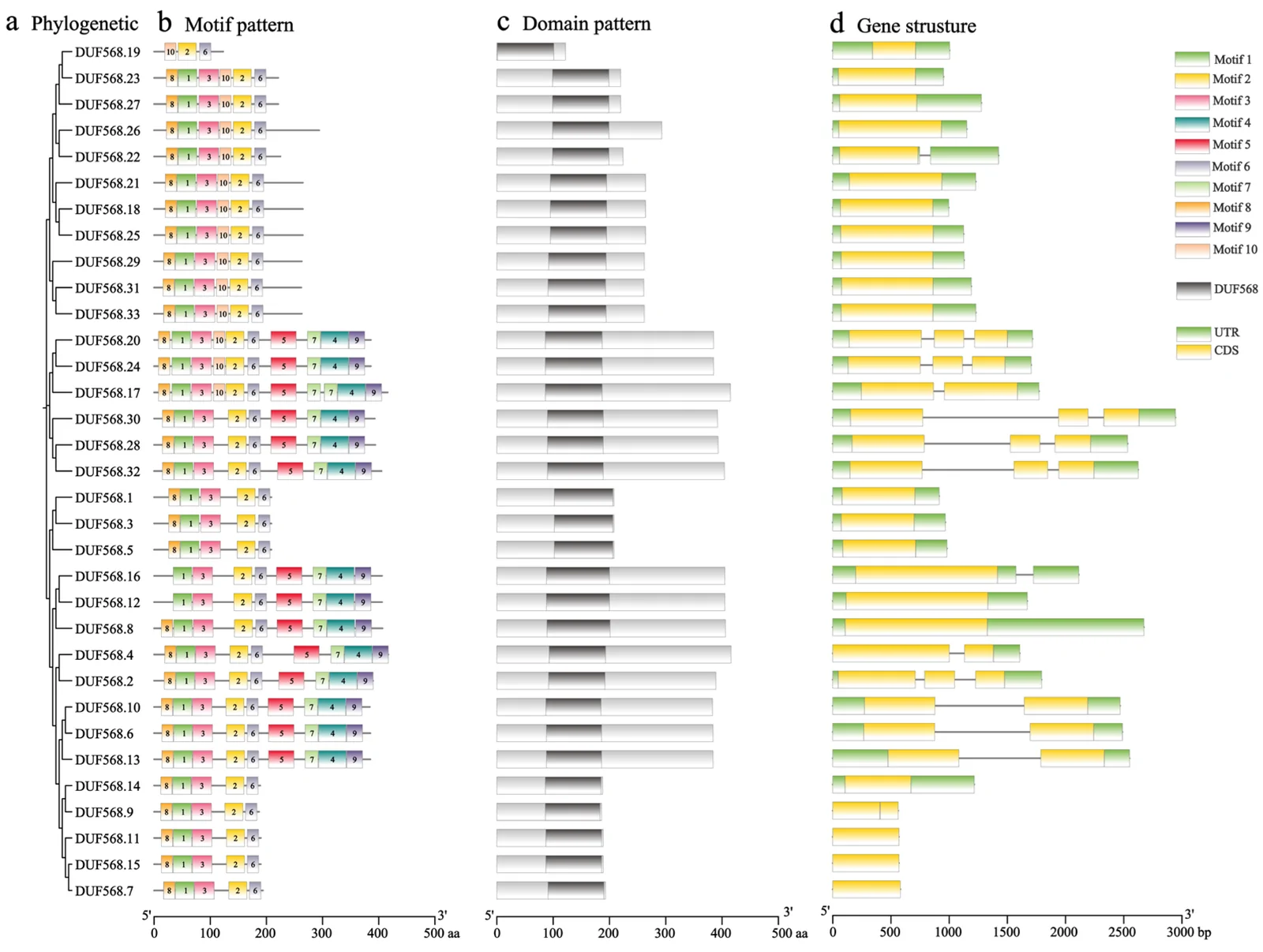
The analysis of the gene structure and protein sequence in wheat DUF568 gene family members: (**a**) the phylogenetic tree of TaDUF568 proteins; (**b**) the motif compositions of TaDUF568 proteins. Motifs 1–10 are displayed in different colored boxes, and the scale at the bottom indicates the length of proteins; (**c**) the domain patterns of TaDUF568 proteins; the DUF568 domains are highlighted in gray; (**d**) exon–intron structures of *TaDUF568* genes, where yellow boxes indicate 5′- and 3′-untranslated regions, green boxes indicate exons, and black lines indicate introns. The horizontal scale bar at the bottom represents sequence length.

To comprehensively characterize the structural features of wheat DUF568 family genes, we analyzed conserved motifs, domain architecture, and exon–intron organization (Figure 2). Ten conserved distinct motifs (Motifs 1–10) were identified. Motifs 1, 2, 3 and 6 were present in the majority of proteins, forming a core conserved module. Notably, genes clustered in the same phylogenetic clade exhibited highly similar motif compositions, suggesting functional conservation within clades.

Domain analysis confirmed that all identified genes contained the canonical DUF568 domain, consistent with their classification into this family. Exon–intron structure analysis revealed variations in intron numbers and gene length among members. Genes in the upper clades generally showed simpler structures with fewer introns, while several genes contained additional exons (e.g., *DUF568.20*, *DUF568.30*) and longer UTR regions (e.g., *DUF568.8*). These structural variations may contribute to functional divergence within the family.

### 2.4. Genome Collinearity Analysis

**Figure 3:**
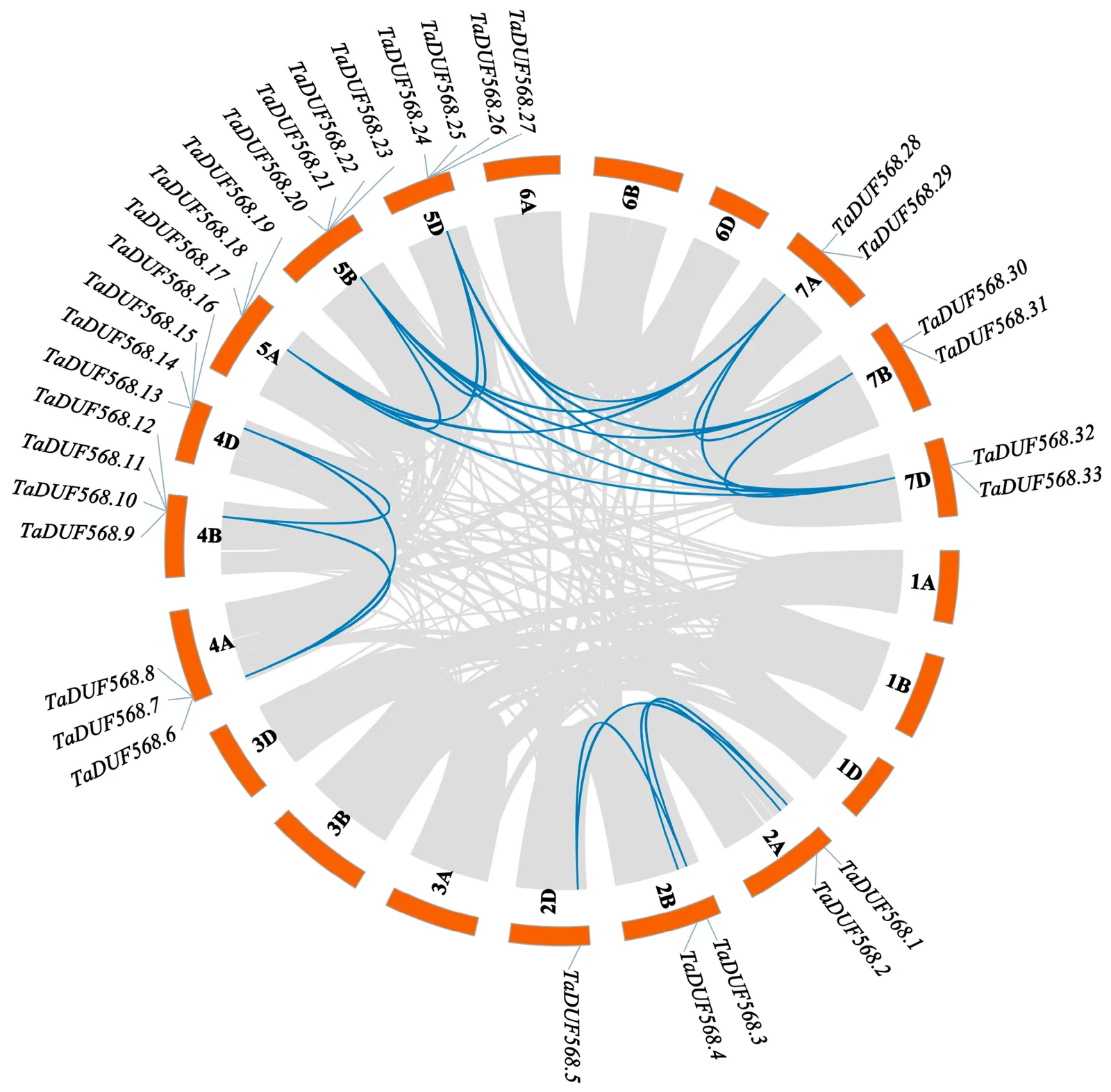
Chromosome localization and collinearity analysis of *DUF568* gene family members in wheat. The gray lines indicate all duplicated gene pairs in the wheat genome; the highlighted blue lines indicate duplicated *TaDUF568* gene pairs.

To investigate the evolutionary relationships of *TaDUF568* genes within the wheat genome, we performed a collinearity analysis across wheat chromosomes (Figure 3). A total of 38 segmental duplication gene pairs were identified, while no tandem duplication events were detected (Table S2). These results indicate that segmental duplication represents the major driving force underlying the expansion of the *TaDUF568* family. These collinear gene pairs were distributed across 12 wheat chromosomes, with high collinearity observed among homologous chromosomes (e.g., chromosomes 2A, 2B and 2D), suggesting conserved evolution of the *DUF568* family in wheat.

**Figure 4:**
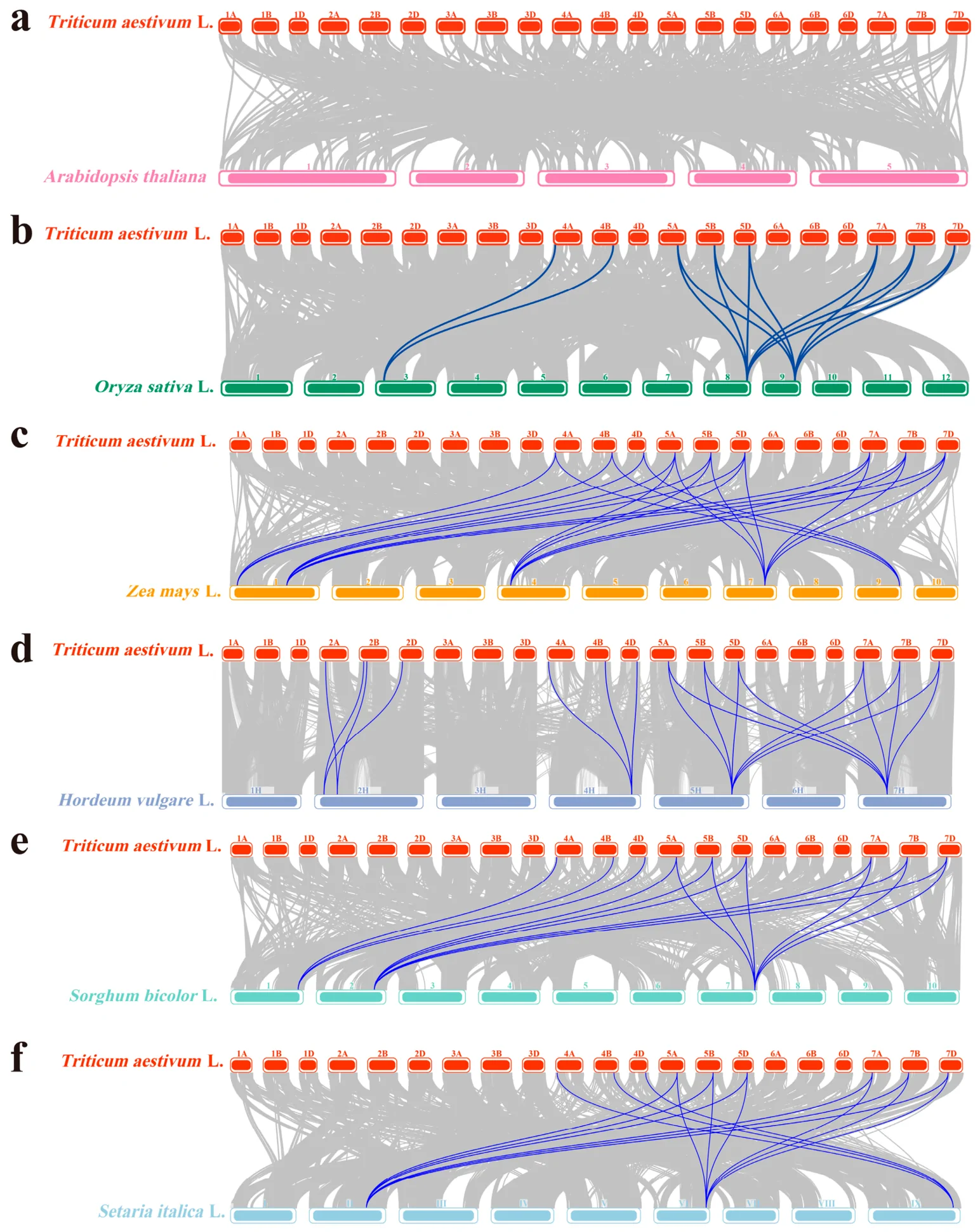
Evolutionary analysis of the wheat *DUF568* gene family members between wheat and other species: (**a**) the syntenic DUF568 gene pairs between *T. aestivum* L. and *A. thaliana*; (**b**) the syntenic DUF568 gene pairs between *T. aestivum* L. and *O. sativa* L.; (**c**) the syntenic DUF568 gene pairs between *T. aestivum* L. and *Z. mays* L.; (**d**) the syntenic DUF568 gene pairs between *T. aestivum* L. and *H. vulgare* L.; (**e**) the syntenic DUF568 gene pairs between *T. aestivum* L. and *S. bicolor* L.; (**f**) the syntenic DUF568 gene pairs between *T. aestivum* L. and *S. italic* L. Gray lines in the background indicate the collinear gene pairs between wheat and the other genome, while the highlighted blue lines represent the syntenic *DUF568* gene pairs.

To explore the evolutionary conservation of the *DUF568* family, interspecies collinearity analysis was performed between wheat and six representative plant species (Figure 4). No collinear *DUF568* gene pairs were detected between wheat and Arabidopsis thaliana. In contrast, 14, 43, 24, 29, and 21 collinear pairs were identified between wheat and rice, maize, barley, sorghum and millet, respectively (Table S3). The highest number of collinear pairs occurred between wheat and maize, reflecting the closer evolutionary relatedness within monocots. Notably, several wheat *TaDUF568* genes showed conserved synteny with multiple orthologous genes in cereal crops, indicating functional conservation of the *DUF568* family during grass evolution.

### 2.5. Expression Pattern Analysis of TaDUF568 Genes

**Figure 5:**
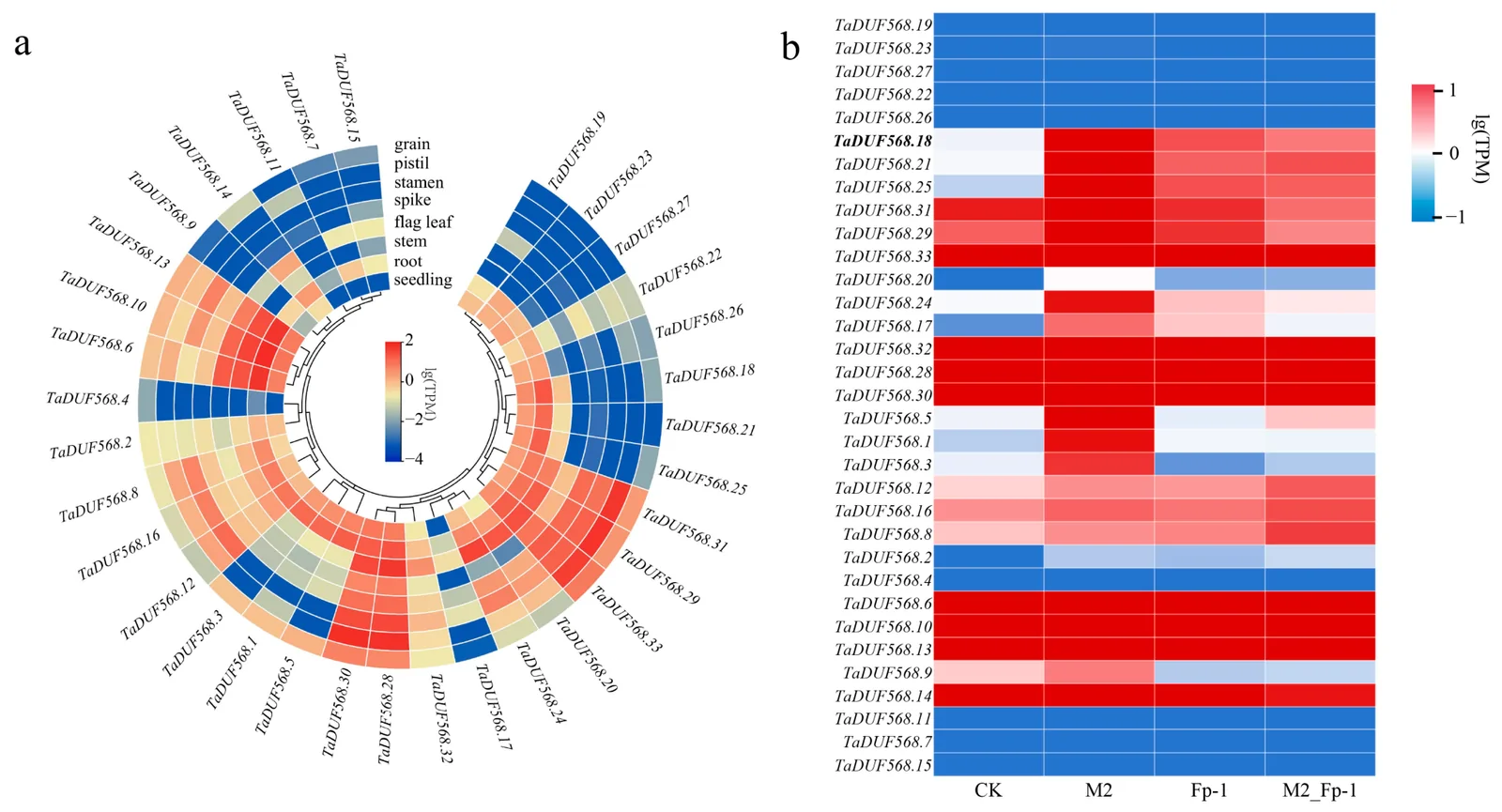
The gene expression profile of the wheat *DUF568* genes: (**a**) expression profiles of wheat DUF568 genes in various organs or tissues of wheat. (**b**) the expression changes in wheat DUF568 genes in different experimental treatments in RNA-seq.

To investigate the spatial expression patterns of TaDUF568 genes, we analyzed their transcriptional levels in eight wheat tissues using publicly available RNA-seq data (Figure 5a). Most TaDUF568 genes exhibited distinct tissue-specific expression patterns, and homologous genes displayed similar expression patterns. Some genes (e.g., *TaDUF568.7*, *TaDUF568.11*, *TaDUF568.17*) showed constitutively low expression across all examined tissues, while *TaDUF568.29*, *TaDUF568.31*, and *TaDUF568.33* were preferentially expressed in reproductive tissues (grain, stamens, and pistils). Additionally, several *TaDUF568* genes exhibited higher transcriptional abundances in roots and seedlings (e.g., *TaDUF568.6*, *TaDUF568.10*, and *TaDUF568.13*; *TaDUF568.18*, *TaDUF568.21*, and *TaDUF568.25*). These divergent expression patterns reflect the functional differentiation of this gene family during tissue development.

RNA-seq was applied to explore transcriptional changes in TaDUF568 genes under four experimental treatments (CK, M2, Fp-1, and M2_Fp-1) to uncover their potential roles in biotic stress responses (Figure 5b). Compared with CK, most genes were significantly upregulated upon M2, Fp-1, and M2_Fp-1 treatments. *TaDUF568.18* displayed the strongest induction after *Trichoderma* M2 treatment (Figure S1), with markedly elevated transcript levels in the M2, Fp-1, and combined M2_Fp-1 groups. Genes including *TaDUF568.21*, *TaDUF568.25*, and *TaDUF568.31* also exhibited consistent upregulation under all three stress treatments. Several genes (e.g., *TaDUF568.19*, *TaDUF568.7*, *TaDUF568.15*) maintained low expression levels in all treatments.

In conclusion, these results indicate that the *TaDUF568.18* gene exhibits preferential expression in wheat roots, and more importantly, its expression is most significantly induced by *Trichoderma* M2 treatment. Transcriptional abundance of *TaDUF568.18* was significantly increased in the M2, Fp-1, and M2_Fp-1 treatment groups. These expression characteristics suggest that *TaDUF568.18* is a promising candidate gene for improving wheat disease resistance upon *Trichoderma* induction, laying a solid foundation for further research on its functions in wheat–microbe interactions and biotic stress responses.

### 2.6. Subcellular Localization of TaDUF568.18 Protein

Based on RNA-seq screening, we identified *TaDUF568.18*, a key gene responsive to *Trichoderma* induction, and successfully cloned this gene. Sequence analysis revealed that this gene has three independent homologous copies, each with a sequence length of 794 bp. Sequence identity among the three homologous copies reaches 98.62% at the nucleic acid level (Figure S2), and 97.98% at the protein level (Figure S3). The core DUF568 domain sequence shows even higher conservation, suggesting that these homoeologs may retain disease resistance-related functions via functional redundancy or subfunctionalization during evolution.

**Figure 6:**
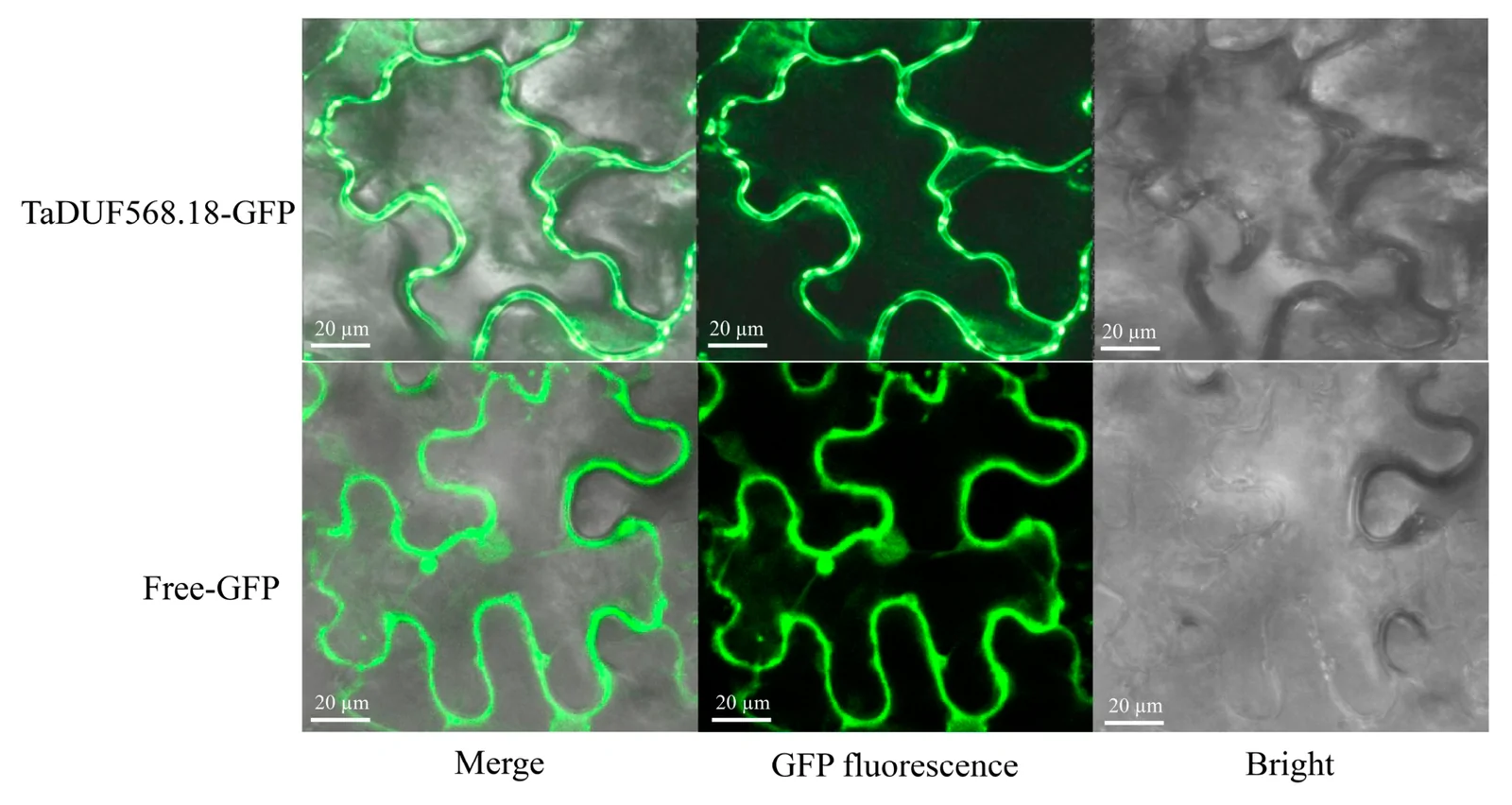
Observation of the subcellular localization of TaDUF568.18 protein in tobacco leaves.

We further constructed a 35S::TaDUF568.18-GFP fusion expression vector and performed subcellular localization analysis using an *Agrobacterium*-mediated transient transformation system in tobacco leaves. Fluorescence microscopy showed that the fluorescence signal of the Free-GFP control was diffusely distributed throughout the cytoplasm and nucleus. In contrast, the green fluorescence signal of TaDUF568.18-GFP specifically accumulated at the cell membrane. This result demonstrates that TaDUF568.18 localizes to the plasma membrane (Figure 6). We speculate that TaDUF568.18 participates in the perception of *Trichoderma*-derived inductive signals and early transmission of anti-pathogen signals at the cell membrane, providing critical subcellular experimental evidence for dissecting its spatial function in *Trichoderma*-mediated resistance against Fusarium crown rot.

### 2.7. Validation of TaDUF568.18 Gene Function in Disease Resistance

**Figure 7:**
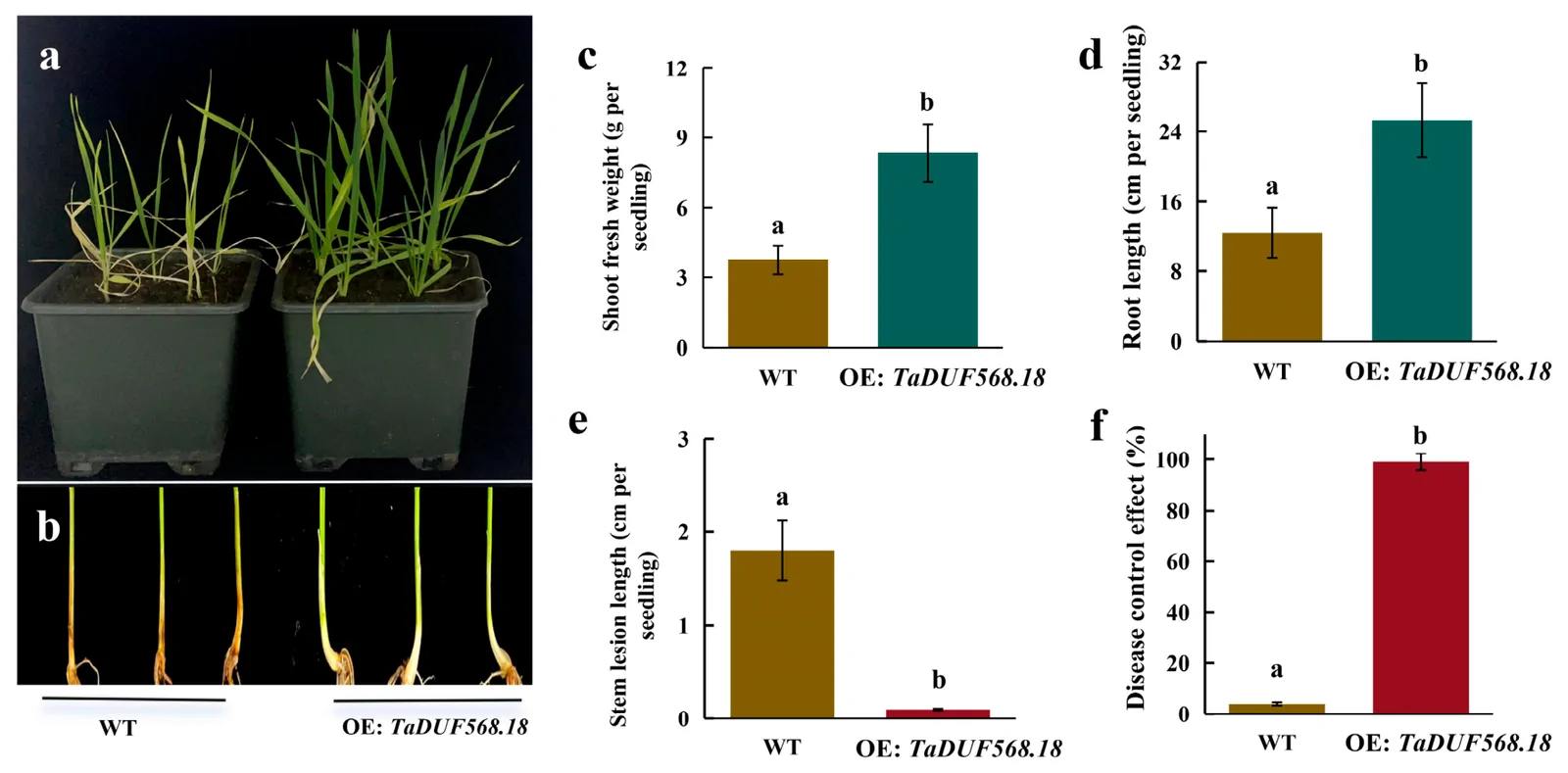
Phenotypic analysis of *TaDUF568.18* overexpression transgenic plants: (**a**) comparison of plant morphology between wild-type and transgenic wheat plants (OE: *TaDUF568.18*); (**b**) effect diagram after pathogen infection at the base of wheat stems; (**c**) fresh weight comparison; (**d**) root length comparison chart; (**e**) comparison of infection length of disease spots at the base of wheat stems; (**f**) comparison of disease control effect at wheat stem bases. Each experiment has three biological replicates. Error bars represent the standard error of means. Different letters at the top of the bars indicate significance (*p* < 0.05; Student’s *t*-test).

Gain-of-function assays were further conducted using *TaDUF568.18*-overexpressing (OE) transgenic wheat lines (Figure 7a). Lesion development was obviously restricted in OE plants compared with WT after pathogen treatment (Figure 7a,b). The overexpression lines showed vigorous growth with robust leaves and enhanced tiller capacity, and no obvious growth suppression was observed under disease stress. Agronomic trait measurement revealed that OE plants possessed a significantly higher fresh weight and longer root length than WT (Figure 7c,d). Moreover, the length of lesions at the basal stem was distinctly reduced in transgenic lines (Figure 7e), and their tolerance to wheat Fusarium crown rot was substantially improved (Figure 7f). These results confirmed a positive correlation between *TaDUF568.18* overexpression and enhanced disease resistance in wheat. Collectively, the evidence from gene overexpression assays demonstrates that *TaDUF568.18* positively modulates both plant growth and defense responses against pathogens in wheat.

## 3. Discussion

### 3.1. Genomic Characteristics and Evolutionary Dynamics of the TaDUF568 Gene Family

In the present study, a total of 33 *DUF568* family genes were identified across the hexaploid wheat genome. The diverse physicochemical properties strongly implied functional differentiation within this gene family, which is a common feature of DUF-containing gene families in higher plants [25,26]. Similar uneven chromosomal distribution and protein property divergence have been reported in wheat *DUF668* and *DUF966* families, whose members also perform diverse biological functions during growth and stress adaptation [27,28]. The phylogenetic tree revealed that Group I is a monocot-specific clade without *A. thaliana* DUF568 homologs, whereas dicot members only occurred in Groups II and III. This pattern agrees with previous reports in rice [10] and maize [29]. Importantly, our functionally characterized gene *TaDUF568.18* falls within this monocot-specific Group I, implying its potential monocot-unique adaptive roles in defense responses. Segmental duplication, rather than tandem duplication, appears to dominate *TaDUF568* family expansion, an evolutionary pattern shared by many disease-resistant gene families in wheat and other cereal crops [30,31]. Collinearity and gene-structure evidence further underscores deep evolutionary conservation of DUF568 within grass lineages, alongside substantial divergence between monocots and eudicots [32]. Congruence between phylogeny, motif composition and exon-intron organization reinforces the link between gene structural features and biological function [33]. Collectively, the genome-wide characterization of *TaDUF568* systematically reveals its evolutionary trajectory, and provides a solid theoretical basis for subsequent functional characterization of key family members involved in biotic stress.

### 3.2. Tissue Specificity and Stress-Responsive Expression of TaDUF568 Family Genes

Distinct tissue-dependent expression among *TaDUF568* paralogs reflects ongoing functional differentiation during wheat organogenesis and tissue specialization, a frequently observed feature in large plant gene families [27,34]. Several paralogs were preferentially expressed in reproductive tissues (grains, stamens, pistils), seedling tissues and roots, implying their potential dual functions in modulating plant development and sensing rhizosphere environmental cues (e.g., microbial signals and nutrient availability) [35]. As the primary interface between plants and soil-borne microbes and pathogens, roots constitutively accumulate a large set of defense-related transcripts to activate early immune surveillance and mount rapid responses to biotic stresses [36,37]. Notably, *TaDUF568.18* and its homeologs are among the most strongly induced family members following challenge by biocontrol *Trichoderma* M2 and the crown rot pathogen *F. pseudograminearum*, pointing to their central involvement in biotic-stress transcriptional reprogramming. As well-established beneficial biocontrol fungi, *Trichoderma* species are capable of activating host-induced systemic resistance and reprogramming the expression of immune-associated genes to enhance plant defense against pathogens [38,39,40,41]. Plasma membrane-resident proteins act as core signal sensors and transducers, which recognize extracellular microbial elicitors (e.g., *Trichoderma*-derived secondary metabolites and pathogen-associated molecular patterns) and initiate downstream immune signaling cascades [42,43,44]. Combined with its root-enriched expression pattern and striking responsiveness to *Trichoderma* induction, the protein TaDUF568.18 was defined as a central regulator governing *Trichoderma*-triggered defense responses in wheat. These findings provide a solid basis for further mechanistic investigation of this key gene and its role in mediating plant–microbe interactions.

### 3.3. TaDUF568.18 Positively Regulates Wheat Resistance Against Soil-Borne Diseases

To validate the biological function of *TaDUF568.18* in wheat disease resistance, we integrated stable transgenic overexpression assays, which are reliable and widely used approaches for gene function verification in wheat [45]. Functional characterization via gene overexpression demonstrated that *TaDUF568.18* positively regulates wheat resistance to *F. pseudograminearum*, the pathogen that causes crown rot and stem base rot, a destructive soil-borne disease responsible for substantial global yield losses [46,47]. Consistently, overexpression of this gene conferred enhanced disease resistance, confirming that *TaDUF568.18* acts as a positive regulator of wheat defense responses [48,49]. Numerous studies have proven that DUF domain-containing proteins broadly participate in plant defense against fungal pathogens across diverse crop species, including rice, maize and barley [50,51,52]. Unlike conventional defense genes that frequently trigger growth–defense trade-offs (e.g., enhanced resistance accompanied by growth inhibition), *TaDUF568.18* concurrently enhances disease resistance and improves agronomic performance, which is a highly desirable trait for modern crop molecular breeding [53]. These findings advance the functional annotation of poorly characterized DUF family members in wheat and extend our mechanistic understanding of *Trichoderma*-triggered plant immunity. Furthermore, *TaDUF568.18* provides a promising gene resource for breeding wheat varieties with improved tolerance to Fusarium crown rot, with practical implications for sustainable wheat production.

### 3.4. TaDUF568.18 (Annotated as TaAIR12), a Resistance Gene with Great Application Potential

Sequence alignment against the Uniprot database (https://www.uniprot.org/) verified that *TaDUF568.18* corresponds to *TaAIR12*, an auxin-induced root culture protein belonging to the DUF568 family. AIR12 was first identified in *Arabidopsis*, and predicted to be a glycosylphosphatidylinositol-anchored protein [14]. Previous studies have indicated that wheat TaAIR12 participates in auxin synthesis, transport, signaling and metabolism [14,15]. The expression of *TaAIR12* is also linked to extracellular redox processes [14], while its full physiological and molecular functions remain poorly understood. In this study, we demonstrated that *TaAIR12* (*TaDUF568.18*) functions as a core positive regulator of *Trichoderma*-triggered defense against soil-borne pathogens, endowing it with great application potential in wheat resistance breeding. Consistent with reported characteristics of *Arabidopsis AIR12* gene [14], *TaDUF568.18* localizes to the plasma membrane, a pivotal site for sensing microbial signals and initiating immune cascades [54]. Multiple studies have proven that DUF568 homologs in cereal crops respond actively to diverse stresses [12,16,55]. Combined with previous reports, we infer that *Trichoderma* M2 triggers auxin signaling reconfiguration to activate *TaAIR12*, which further modulates extracellular ROS balance and systemic resistance [14]. More importantly, *TaAIR12* simultaneously improves disease resistance and plant growth, avoiding the typical growth–defense trade-off. As the first functionally characterized *TaAIR12* involved in *Trichoderma*-mediated plant immunity, this gene fills the research gap of *DUF568* in crop biotic stress and serves as a superior candidate gene for wheat molecular resistance breeding.

## 4. Materials and Methods

### 4.1. Plant and Microbial Materials

The wheat cultivar *Triticum aestivum* cv. Zhengmai 1860, *Trichoderma harzianum* M2 (biocontrol strain), and *Fusarium pseudograminearum* (Fp) strain Fp-1 (wheat Fusarium crown rot pathogen) were used in this study and preserved in our laboratory. Microbial strains were routinely cultured on potato dextrose agar (PDA). *T. harzianum* M2 was incubated at 28 °C for 7 d to harvest conidia, while *F. pseudograminearum* Fp-1 was cultured at 25 °C for 10 d. Conidial suspensions were adjusted to 1 × 10^7^ spores/mL for M2 induction and Fp-1 inoculation, respectively. Wheat seeds were surface-sterilized and germinated in a controlled growth chamber (25 °C, 16 h light/8 h dark cycle, 70% relative humidity) prior to cultivation in vermiculite.

### 4.2. RNA-Seq and Sample Preparation

Four independent treatment groups were established in this study: blank control (CK, sterile water treatment), only M2 induction (M2), single Fp-1 infection (Fp-1), and combined M2 induction followed by Fp-1 infection (M2_Fp-1). For the M2 treatment, wheat seedling roots were irrigated with M2 conidial suspension; for Fp-1 treatment, seedlings were inoculated with Fp-1 spore suspension; for the M2_Fp-1 group, Fp-1 pathogen inoculation was performed 24 h after M2 induction. Wheat root tissues were sampled at 48 h post-treatment with three biological replicates. Total RNA was extracted using Trizol reagent, and subsequently used for cDNA library construction for RNA-seq. RNA-seq was performed on the Illumina NovaSeq 6000 platform. Clean reads were mapped to the wheat reference genome IWGSC RefSeq v1.1 (http://plants.ensembl.org/). The raw transcriptome data are deposited in the NCBI (https://www.ncbi.nlm.nih.gov) under the project number PRJNA1247731.

### 4.3. Identification and Phylogenetic Analysis of DUF568 Gene Family Members

The DUF568 HMM profile (PF04526) [10] was retrieved from Pfam (http://pfam.xfam.org/ (accessed on 15 March 2026)) for genome-wide gene screening in wheat, *Arabidopsis*, rice, maize, barley, sorghum, and millet with an E-value cutoff of 1 × 10^−10^. Candidate genes were further verified using SMART (http://smart.embl-heidelberg.de/ (accessed on 15 March 2026)) and NCBI CDD (https://www.ncbi.nlm.nih.gov/Structure/bwrpsb/bwrpsb.cgi/ (accessed on 15 March 2026)) to retain sequences with complete DUF568 domains. Genomic sequences of the seven species were downloaded from Ensembl Plants (https://www.ensembl.org/ (accessed on 15 March 2026)). Full-length protein sequences were aligned using MEGA v7.0 (http://www.megasoftware.net/), and a neighbor-joining phylogenetic tree was constructed using IQ-TREE v2.4.0 (http://www.iqtree.org/ (accessed on 15 March 2026)) with 1000 bootstrap replicates and visualized using software FigTree v1.4.4 (https://tree.bio.ed.ac.uk/software/figtree/ (accessed on 15 March 2026)).

### 4.4. Sequence Analysis of DUF568 Gene Family Members

Exon–intron structures were extracted from the wheat genome annotation file. Conserved protein motifs were predicted using MEME Suite (https://meme-suite.org/meme/tools/meme (accessed on 15 March 2026)), with parameters configured to identify up to 10 conserved motifs. All structural and element features were visualized and summarized using the software TBtools v2.390 [56].

### 4.5. Synteny Analysis of DUF568 Gene Family Members

Intraspecific and interspecific synteny analyses were performed using MCScanX implemented in the software TBtools v2.390 (https://www.github.com/CJ-Chen/TBtools, accessed on 15 March 2026). BLASTP searches (E-value < 1 × 10^−10^) were conducted to identify homologous gene pairs between wheat and the other six species. Gene duplication events and collinear relationships were visualized to evaluate the evolutionary conservation and divergence of the DUF568 gene family using the software TBtools v2.390 [56].

### 4.6. Gene Expression Profiling

Expression patterns of *TaDUF568* genes were analyzed based on public GEO transcriptome data (http://www.wheat-expression.com/ (accessed on 15 March 2026)), and RNA-seq data under *Trichoderma* induction and pathogen infection. The TPM (Transcripts per million) values have been normalized to reflect the relative levels of gene expression. Heatmaps showing gene expression levels were generated using the software TBtools v2.390 to screen core *Trichoderma*-responsive candidate genes [56]. The detailed methods for RNA-seq and related bioinformatics analysis can be found in a previous study [56].

### 4.7. Subcellular Localization

The full-length coding sequence of *TaDUF568.18* lacking the stop codon was amplified from wheat root cDNA using specific primers. The amplified fragment was inserted into the pCAMBIA1304-35S-GFP vector to generate the 35S::*TaDUF568.18*-GFP fusion construct. The recombinant plasmid and empty vector were separately transformed into *Agrobacterium tumefaciens* strain GV3101. Bacterial cultures were resuspended in infiltration buffer and infiltrated into epidermal cells of *Nicotiana benthamiana* leaves. Then, GFP fluorescence signals were observed and imaged using a laser confocal scanning microscope. The subcellular distribution of *TaDUF568.18* was determined by comparing the fluorescence pattern of the fusion protein with the empty GFP control. The primer sequences used in the experiment are shown in Table S4.

### 4.8. Overexpression Vector Construction and Generation of Transgenic Wheat

The full-length coding sequence of *TaDUF568.18* was amplified from wheat root cDNA using gene-specific primers. The amplified fragment was ligated into a plant overexpression vector driven by the cauliflower mosaic virus 35S promoter. The recombinant construct was transformed into *A. tumefaciens* strain EHA105, which was subsequently used for the genetic transformation of wheat immature embryos via the *Agrobacterium*-mediated method. Positive transgenic lines were screened by PCR and qRT-PCR, and homozygous *TaDUF568.18*-overexpression lines were selected for subsequent phenotypic assays. WT wheat plants were used as the control throughout the experiments.

All phenotypic and physiological measurements were performed on wheat plants at the tillering stage. After 14 days of pathogen inoculation, the basal stem lesion length of OE and WT plants was measured. Plant fresh weight and root length were determined with conventional morphological detection methods under consistent growth conditions. The disease control effect was calculated based on the lesion reduction rate of transgenic lines relative to WT plants, which differs from the disease index: the disease index reflects the overall disease severity of plant populations, while the disease control effect intuitively evaluates the degree of improvement in disease resistance in OE: *TaDUF568.18* wheat [57]. All phenotypic data were obtained from three independent biological replicates to ensure experimental accuracy.

### 4.9. Pathogen Inoculation and Phenotypic Evaluation

Uniform seedlings of WT and transgenic wheat lines were grown under consistent culture conditions. At the designated growth stage, seedlings were inoculated with the causal pathogen Fp-1. Plant growth performance was regularly monitored during the experimental period. At the set time points, leaf morphology and tiller status were recorded. Plant fresh weight and root length were measured to evaluate vegetative growth traits. The lesion length at basal stems was quantified, and the disease resistance level of each group was statistically assessed. Disease index (DI) = 100 × ∑ (number of investigated single plants × representative value of each plant)/(total number of investigated plants × highest representative value). Disease control effect (%) = [(DI_MOCK_ − DI_OE_)/DI_MOCK_] × 100%. For detailed description methods, refer to a previous study [58]. All experiments were performed with three biological replicates to ensure the reliability of results.

### 4.10. qRT-PCR and Statistical Analysis

qRT-PCR was conducted using the SYBR Green qPCR Master Mix with wheat *Actin* gene as the internal reference. A detailed description is provided in a previous study [58]. The primer sequences used in the experiment are shown in Table S4. All phenotypic and molecular data were analyzed by one-way ANOVA with Duncan’s multiple range test (*p* < 0.05) using SPSS v26.0 (https://www.ibm.com/products/spss-statistics/ (accessed on 15 March 2026)). Relative expression levels were calculated via the 2^−ΔΔCt^ method with three biological and technical replicates [59]. Each treatment included three independent biological replicates. Data are presented as mean ± standard error. Statistical analyses and mapping were completed with GraphPad Prism v10 (www.graphpad.com).

## 5. Conclusions

This study comprehensively characterized the *TaDUF568* gene family in wheat, identifying 33 members with distinct genomic and structural characteristics. The *TaDUF568.18* encodes a plasma membrane-localized protein and was specifically induced by *Trichoderma* M2. Functional experiments demonstrated that *TaDUF568.18* (annotated as *TaAIR12*) positively regulate wheat resistance to Fusarium crown rot and can coordinate plant growth and defense responses. This is the first report demonstrating that a DUF568 gene mediates *Trichoderma*-induced defense responses in wheat, enriching the functional annotation of the DUF gene family in wheat and providing valuable candidate genes for improving wheat resistance against Fusarium crown rot.

## Figures and Tables

**Table 1:** Wheat DUF568 gene family members.

Gene Name	Genomic Accession	Chromosome	Physical Position	Protein Prediction		
				Amino Acid (aa)	Molecular Mass (kD)	Isoelectric Point
*TaDUF568.1*	TraesCS2A02G129200.1	2A	77321064..77321978	208	21.23	6.26
*TaDUF568.2*	TraesCS2A02G190300.1	2A	156680227..156682021	389	41.48	9.34
*TaDUF568.3*	TraesCS2B02G151500.1	2B	118311639..118312606	208	21.22	6.57
*TaDUF568.4*	TraesCS2B02G216700.1	2B	202904168..202905775	416	44.85	8.95
*TaDUF568.5*	TraesCS2D02G131800.1	2D	76801794..76802775	208	21.17	7
*TaDUF568.6*	TraesCS4A02G044000.1	4A	36611905..36614393	384	40.45	10.12
*TaDUF568.7*	TraesCS4A02G044100.1	4A	36794778..36795359	193	19.51	9.39
*TaDUF568.8*	TraesCS4A02G044400.1	4A	37006921..37009594	406	42.44	9.68
*TaDUF568.9*	TraesCS4B02G264900.1	4B	535747341..535747903	186	19.08	6.7
*TaDUF568.10*	TraesCS4B02G265100.1	4B	536117744..536120213	383	40.59	10.01
*TaDUF568.11*	TraesCS4B02G265200.1	4B	536412001..536412570	189	19.40	9.18
*TaDUF568.12*	TraesCS4B02G265500.1	4B	536894668..536896339	405	42.45	9.68
*TaDUF568.13*	TraesCS4D02G265000.1	4D	435207279..435209829	384	40.36	9.98
*TaDUF568.14*	TraesCS4D02G265100.1	4D	435230622..435231838	188	19.31	6.24
*TaDUF568.15*	TraesCS4D02G265200.1	4D	435410559..435411128	189	19.21	8.74
*TaDUF568.16*	TraesCS4D02G265500.1	4D	435626783..435628897	405	42.36	9.68
*TaDUF568.17*	TraesCS5A02G278800.1	5A	488022784..488024556	415	43.26	9.46
*TaDUF568.18*	TraesCS5A02G278900.1	5A	488038395..488039391	264	26.09	9.19
*TaDUF568.19*	TraesCS5A02G279000.1	5A	488050904..488051908	122	12.75	5.48
*TaDUF568.20*	TraesCS5B02G278100.1	5B	463992140..463993856	385	39.89	9.43
*TaDUF568.21*	TraesCS5B02G278200.1	5B	464144389..464145617	264	26.21	9.19
*TaDUF568.22*	TraesCS5B02G278300.1	5B	464170972..464172396	224	22.70	6.19
*TaDUF568.23*	TraesCS5B02G278400.1	5B	464195264..464196215	220	22.61	6.14
*TaDUF568.24*	TraesCS5D02G285700.1	5D	386150496..386152200	385	39.64	9.43
*TaDUF568.25*	TraesCS5D02G285900.1	5D	386209719..386210843	264	26.26	9.19
*TaDUF568.26*	TraesCS5D02G286000.1	5D	386512857..386514008	293	29.37	4.78
*TaDUF568.27*	TraesCS5D02G286100.1	5D	386589437..386590714	220	22.64	6.29
*TaDUF568.28*	TraesCS7A02G255600.1	7A	243647433..243649966	393	40.69	9.8
*TaDUF568.29*	TraesCS7A02G255700.1	7A	243694528..243695656	262	25.35	9.7
*TaDUF568.30*	TraesCS7B02G152000.1	7B	202160634..202163579	392	40.76	9.83
*TaDUF568.31*	TraesCS7B02G152200.1	7B	202359388..202360578	261	25.36	9.6
*TaDUF568.32*	TraesCS7D02G254000.1	7D	230814831..230817455	404	42.06	9.71
*TaDUF568.33*	TraesCS7D02G254100.1	7D	230941708..230942937	262	25.37	9.6

## Data Availability

*Trichoderma harzianum* M2 was deposited in the China General Microbiological Culture Collection Center (https://cgmcc.net/). It was originally labeled T-M2 and assigned the preservation number CGMCC NO. 41972. In the present study, M2 and Fp carry GenBank accession numbers PV802261 and PV810307 respectively (https://www.ncbi.nlm.nih.gov/). Raw RNA-seq transcriptomic data are available at NCBI under BioProject PRJNA124773. All original research outputs are provided in the article and Supplementary Files; readers may contact the corresponding author for further questions.

## Supplementary Materials

The following supporting information can be downloaded at: https://www.mdpi.com/article/10.3390/plants15172705/s1, Figure S1: Gene expression analysis of *TaDUF568.18*; Figure S2: Analysis of the nucleic acid sequence of wheat *TaDUF568.18* and homologous genes; Figure S3. Sequence analysis of the wheat TaDUF568.18 and homologous proteins; Table S1: The sequence information used in the phylogenetic tree; Table S2: One-to-one orthologous relationships between wheat and wheat; Table S3: One-to-one orthologous relationships between wheat and other species; Table S4: The primer sequences used in this study.

## Abbreviations

The following abbreviations are used in this manuscript:

At	*Arabidopsis thaliana*
DUF	Domains of unknown function
*Fp*	*Fusarium pseudograminearum*
Hv	*Hordeum vulgare*
OE	overexpression
Os	*Oryza sativa*
PDA	potato dextrose agar
pI	isoelectric point
Sb	*Sorghum bicolor*
Si	*Setaria italica*
Ta	*Triticum aestivum*
TPM	Transcripts per million
WT	Wild-type
Zm	*Zea mays*
