# Supplementary material for: The Key Trichoderma-Induced Gene Encoding a DUF568 Domain-Containing Protein Mediates Defense Responses in Wheat
Source: Plants (Basel). 2026 Sep 3;15(17):2705. doi: 10.3390/plants15172705 (PMC13567248; doi:10.3390/plants15172705)
Supplement: Supplementary file 1 [file plants-15-02705-s001.zip › Supplementary Figures.pdf]

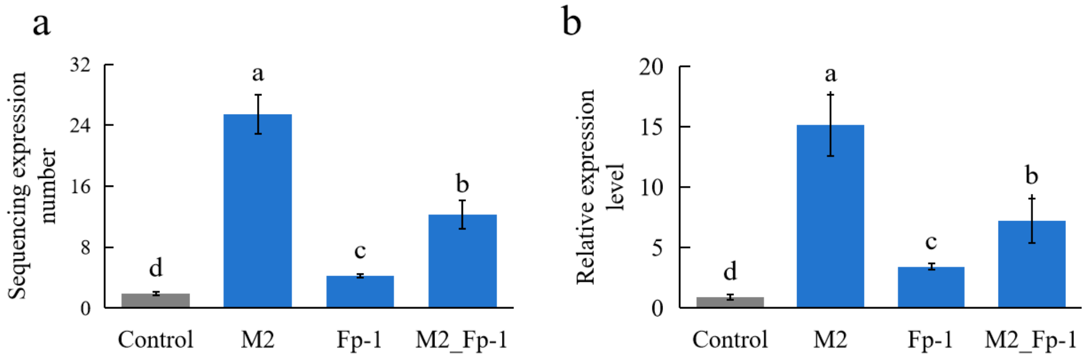


**Figure S1 Gene expression analysis of *TaDUF568.18*.** a. Expression analysis of wheat *TaDUF568.18* gene in RNA-seq; b. RT-PCR analysis of wheat *TaAIR12* gene under different treatments

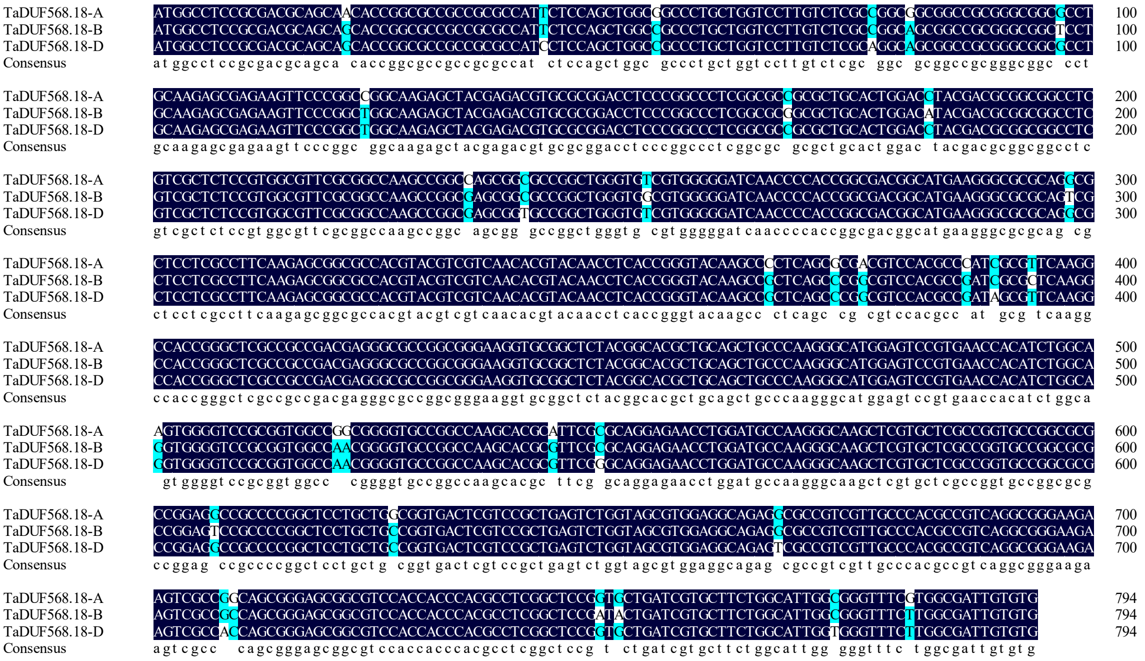


**Figure S2 Analysis of the nucleic acid sequence of wheat *TaDUF568.18* and homologous genes**

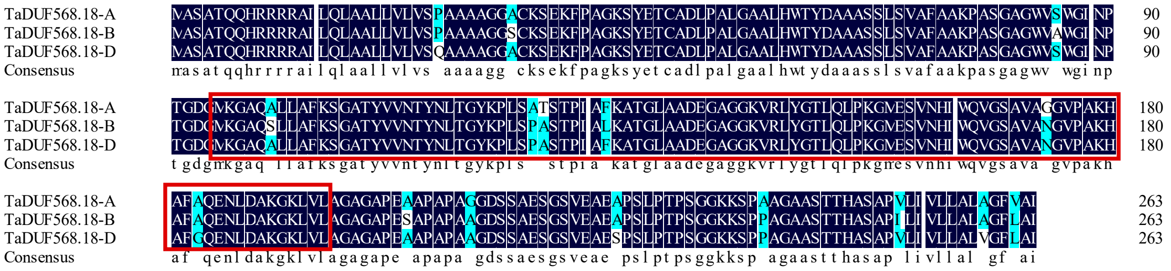


**Figure S3 Sequence analysis of the wheat TaDUF568.18 and homologous proteins.** The red box represents the DUF568 domain region.
